# Choosing between an Apple and a Chocolate Bar: the Impact of Health and Taste Labels

**DOI:** 10.1371/journal.pone.0077500

**Published:** 2013-10-14

**Authors:** Suzanna E. Forwood, Alexander D. Walker, Gareth J. Hollands, Theresa M. Marteau

**Affiliations:** Behaviour and Health Research Unit, University of Cambridge, Cambridge, United Kingdom; Duke University Medical Center, United States of America

## Abstract

Increasing the consumption of fruit and vegetables is a central component of improving population health. Reasons people give for choosing one food over another suggest health is of lower importance than taste. This study assesses the impact of using a simple descriptive label to highlight the taste as opposed to the health value of fruit on the likelihood of its selection. Participants (N=439) were randomly allocated to one of five groups that varied in the label added to an apple: apple; healthy apple; succulent apple; healthy and succulent apple; succulent and healthy apple. The primary outcome measure was selection of either an apple or a chocolate bar as a dessert. Measures of the perceived qualities of the apple (taste, health, value, quality, satiety) and of participant characteristics (restraint, belief that tasty foods are unhealthy, BMI) were also taken. When compared with apple selection without any descriptor (50%), the labels combining both health and taste descriptors significantly increased selection of the apple (’healthy & succulent’ 65.9% and ‘succulent & healthy’ 62.4%), while the use of a single descriptor had no impact on the rate of apple selection (‘healthy’ 50.5% and ‘succulent’ 52%). The strongest predictors of individual dessert choice were the taste score given to the apple, and the lack of belief that healthy foods are not tasty. Interventions that emphasize the taste attributes of healthier foods are likely to be more effective at achieving healthier diets than those emphasizing health alone.

## Introduction

Increasing the consumption of fruit and vegetables (F&V) is a key component of obesity prevention, as their consumption favours a healthy weight [1] and reduces the risks of associated diseases, such as type 2-diabetes [2]. Recent public health campaigns have focused on the health-enhancing qualities of fruit and vegetables over less healthy foods, and encourage a minimum consumption of several portions of fruit and vegetables a day. Such campaigns include the ‘Fruits and Veggies-More Matters’ in the U.S, the UK’s ‘5 A DAY’ campaign, Australia’s ‘Go 2 and 5’ and the French ‘10parjour’ program. 

Although fruit and vegetable consumption is clearly beneficial for health, emphasising their health benefits may not be the most effective strategy. Several studies have shown taste as a primary motive in food choice, with most people viewing health and weight control as lower priorities [3–5]. 

In addition, there is evidence for a commonly held belief in the U.S. population that health and taste are inversely related to each other [6]. Highlighting the health of a food may therefore have the unwanted effect of discouraging people from choosing that food. This is supported by a number of experiments where the perceived healthiness of nutritionally ambiguous foods is inversely related to expected satiety, anticipated pleasure and reported taste of the food [7–9]. Accordingly, labelling food in vending machines as healthy has only a negligible impact on choice and consumption (Horgen & Brownell, 2002).

The aim of the current study was to determine whether using a simple intervention to highlight the tastiness of a fruit increased the choice of fruit over a less healthy alternative. Based on the preceding discussion, it was predicted that highlighting the tastiness of an apple would increase the rate of fruit selection while highlighting the healthiness would have little effect on fruit selection. The simple use of a label is predicted to increase the association between the fruit being labelled and the attribute in the label, so a secondary prediction was that any effects on fruit selection of highlighting tastiness in the label is mediated by the perceived tastiness of the labelled fruit.

The task used in the current study involved participants selecting the components of a ‘combo meal’. The measure of interest was the selection of the dessert, presented as a choice between a chocolate bar and an apple, with the label of the apple being the experimental manipulation. Five labels were used: apple; healthy apple; succulent apple; healthy and succulent apple; succulent and healthy apple. 

As well as assessing the impact of the labelling intervention on apple selection, a secondary analysis sought to explore individual differences in apple selection. To this end, a number of additional measures were assessed. These included ratings for properties associated with the labelled apple that might influence choice (taste, health, value for money, quality, satiety), belief that health and taste seldom co-occur in foods [6], and a range of individual measures such as concern with managing their weight [10], restraint [11], gender, age, BMI, and dieting status. Again, as per the preceding discussion, the primary prediction for this analysis was that individuals that viewed the apple as tasty, or individuals who believed that healthy fruits could also be tasty were more likely to select an apple over a chocolate bar.

## Methods

### Participants

493 participants were recruited using the Amazon Mechanical Turk platform (https://www.mturk.com/mturk/welcome). Participants were recruited on a voluntary basis to take part in a ‘food survey’ and received monetary compensation of $1.10 for completing the 8 minute task. The task was only available to US residents. 

All participants provided written and informed consent to take part in the study before proceeding with any testing, as approved by the ethics committee. Ethical approval was provided by the University of Cambridge Psychology Research Ethics Committee (PRE 2011-57).

### Procedure

The current task was conducted entirely online by participants using web-based recruitment and testing. Following recruitment and consent, participants were randomly allocated to one of five experimental groups.

**Figure 1:**
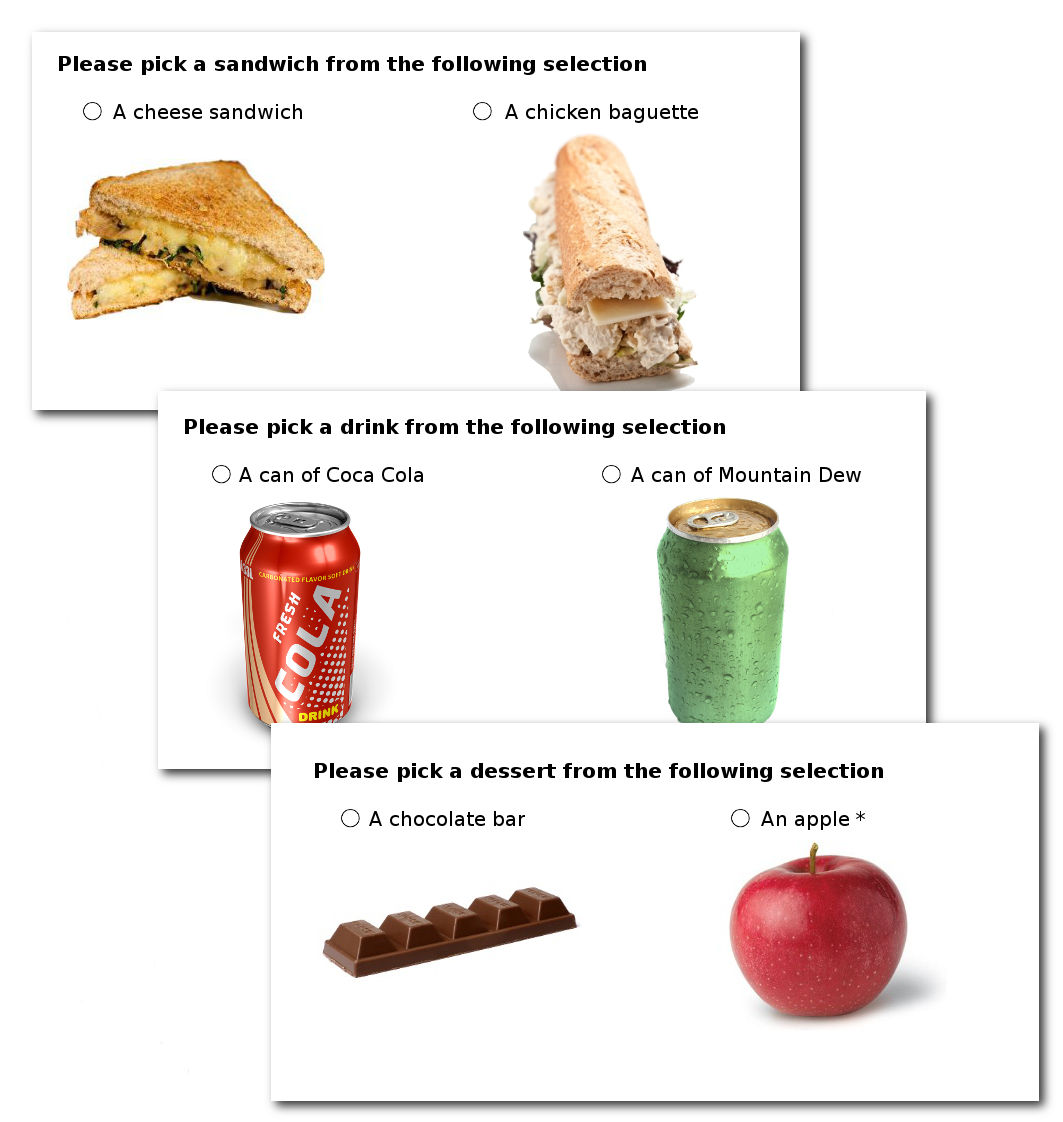
Food images used in the study (drinks for illustration purposes only – participants were shown branded products). Participants were asked to choose the components of a fixed price ‘combo’ meal. All participants were asked to select components in the same order (sandwich, drink, dessert), with the left/right allocation randomised. *The apple label differed by participant group allocation. Alternatives for the four other groups were “a healthy apple”, “a succulent apple”, “a healthy and succulent apple” or “a succulent and healthy apple”.

All participants were first instructed to choose the components of a fixed price ‘combo meal’. They could choose from one of two sandwiches, one of two cans of carbonated sweetened drink and one of two desserts (Figure 1 for images use). For all of the options they were given both a pictorial representation and a prominent verbal description. 

The sandwich and drinks choices were chosen to be realistic options that were similarly low in healthiness and high in taste. The sandwich options were a toasted cheese sandwich pictured with the melted cheese clearly visible and described as “cheese sandwich (cheese, butter, mushroom and watercress, white loaf bread)”, or a chicken salad baguette pictured with the chicken and mayonnaise clearly visible and described as “chicken baguette (chicken salad, cheese and lettuce, baguette)”. The drinks options were images of 12 fluid ounce (355ml) cans of regular Coca-Cola or Mountain Dew and were labelled with the brand and quantity.

The dessert choice was the primary outcome measure in the current study. The choice was between an unambiguously healthy option and an unambiguously unhealthy option: an apple and a chocolate bar. How the apple was labelled differed between groups. For all groups the chocolate bar was labelled as “chocolate bar (Allergy information: nut-free)”. For the control group the apple was labelled as “apple (Origin: U.S.)”. Four further groups saw the same label with additional adjectives in the apple label to emphasise health or taste: “healthy apple”, “succulent apple”, “healthy and succulent apple” or “succulent and healthy apple”.

Following the ‘combo meal’ choice, participants from each group were presented in turn with the chocolate bar and the apple, both labelled as before with additional price information (“Price: $0.70”). They were asked to rate these items on a five-point scale for taste (‘How tasty do you think this food product would be?’), health (‘To what extent do you think this food product is good for you?’), value (‘To what extent do you think this food product is good value for money?’), quality (‘How would you rate the quality of this food product?’), and satiety (‘How filling do you think this food product would be?’).

Participants were next asked to rate their liking on an 8-point scale (‘dislike very much’ to ’like very much’) for a range of food products: five fruit items (strawberries, grapes, fruit salad, oranges, apples) and five relatively unhealthy snack items (ice cream, plain cookies, homemade cake, chocolate bars, chocolate chip cookies). These items were all presented as words, with no pictures. This measure was used to exclude participants who expressed a dislike (i.e. rated as ‘dislike very much’) for apple or chocolate, as these participants were unlikely to have a free choice in the task.

Finally participants were asked the extent to which they were concerned with managing their weight [10], to complete a restraint scale [11], their explicit belief that unhealthy foods are tasty [6], and to provide self-reported gender, age, height, weight, and whether they were presently on a diet to lose weight or maintain their current weight.

### Analysis

#### Effect of labelling intervention on apple selection

Since the measure of interest – choice of dessert – was a binary outcome, a logistic regression model was used. The study design assumed that the choice of sandwich and drink would not influence choice of dessert. To test whether this assumption was in line with the choices made, a regression was first run looking at the impact of sandwich and drink choice on dessert choice.

In order to assess the impact of the label on dessert choice a logistic regression model of dessert choice by label was run. Mediation of any observed effect of labelling was assessed by first testing for an association between the intervention and the proposed mediator, namely perceived tastiness. 

#### Correlational analysis of apple selection

To better understand the factors that might have influenced the decision to choose an apple over a chocolate bar, a forward step-wise method was used to find the strongest predictors of selecting the apple. The factors to be explored in the model include the following categorical variables: the label, participant restraint group (low or high, splitting by midpoint of restraints scale), participant BMI group (lean, overweight, obese), belief that unhealthy foods are tasty (split by response score: agree 2-5, neutral 6, disagree 7-10), as well as continuous variables of the individual ratings given for the five properties of the apple (taste, health, value, quality, satiety). If any factors were found to have a main effect, potential interactions with the label were included into the model.

## Results

Of the 493 respondents, five were rejected for completing the task twice (as determined by IP address and Mechanical Turk worker ID), another 15 were rejected for indicating a strong dislike for either apples (nine respondents) or chocolate bars (six respondents), and two were rejected for having BMIs under 16, indicating potential eating disorders or health problems. This left 471 respondents to include in the final analysis. The mean age of participants was 32.5 (standard deviation 11.6), of whom half were male (n=234), and a mean BMI of 26.4 (standard deviation 7.5).

There was no evidence that choice of sandwich (B=-0.161, p=0.52), drink (B=-0.049, p=0.88) or an interaction between them (B=0.351, p=0.39), affected the choice of dessert: 

### Effect of labelling intervention on apple selection

The rate of apple selection when no descriptive label was present was 50%. The inclusion of a simple label did not alter apple selection rates (‘healthy’ 50.5% and ‘succulent’ 52%), though the inclusion of a combined label did increase selection of the apple (’healthy & succulent’ 65.9% and ‘succulent & healthy’ 62.4%) A logistic regression of dessert choice by label, using the no descriptive label as the reference condition, confirmed this finding (Table 1).

**Table 1:** Logistic regression model assessing the predictors of individual apple choice.

	B	Std. Error	*P*	
Label				
No label	ref			
‘Healthy’	0.02	0.29	0.94	
‘Succulent’	0.08	0.29	0.78	
‘Healthy & Succulent’	0.66	0.30	0.03	*
‘Succulent & Healthy’	0.51	0.30	0.09	

* = p<.05

To assess the hypothesised mediation by perceived tastiness of the observed effect of the combined label, we collapsed the five study groups into a ‘combined label’ group and a ‘simple or no label’ group. We found no significant association between intervention group and perceived tastiness (B=.060, p=0.40), indicating that tastiness was not mediating the observed effect of the intervention on apple choice. 

### Correlational analysis of apple selection

**Figure 2:**
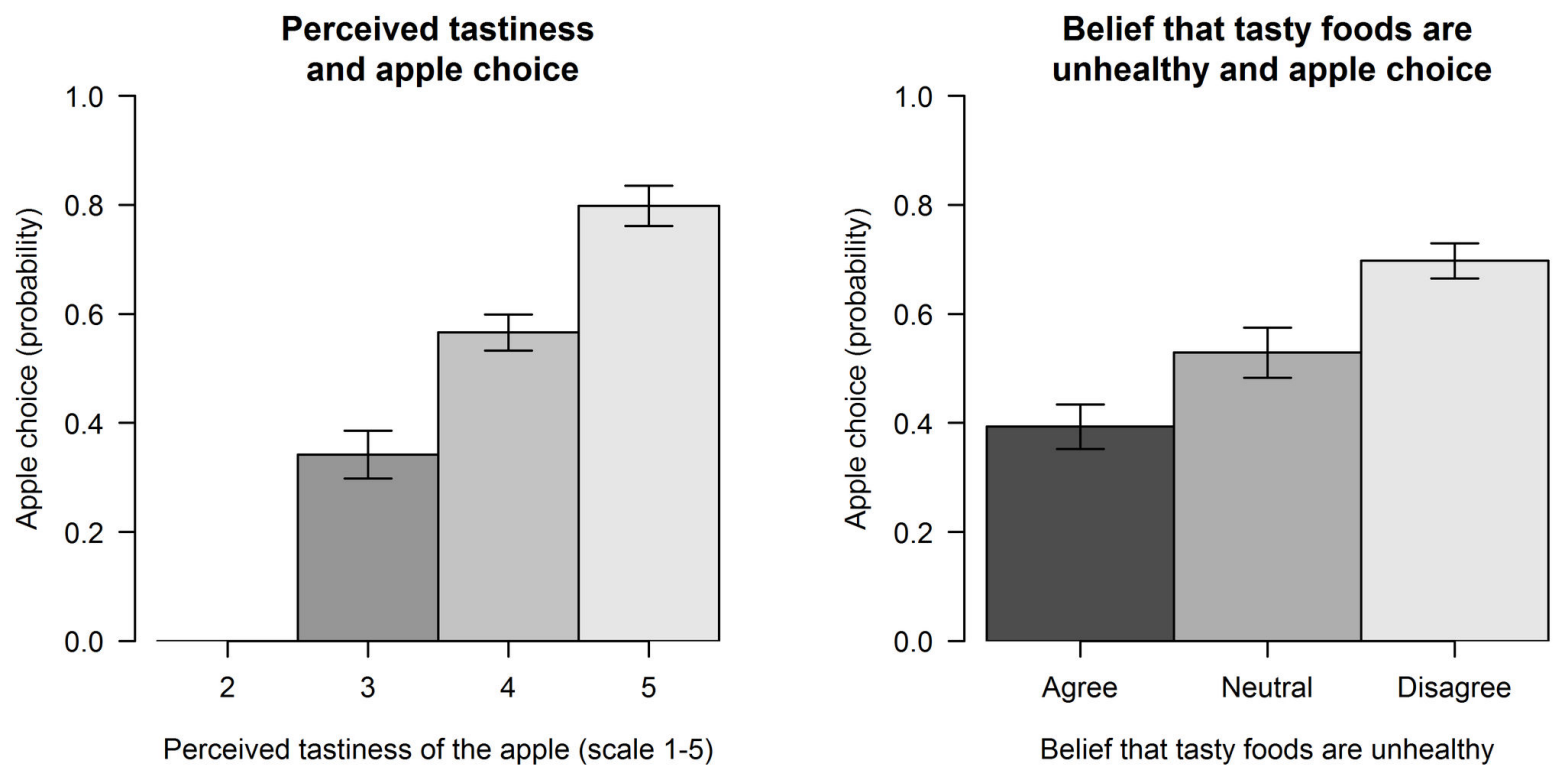
Apple choice and (a) Perceived Tastiness, and (b) Belief that tasty foods are unhealthy.

The forward stepwise regression model identified perceived taste of the apple as the strongest predictor of dessert choice (Figure 2). This was followed by the belief that tasty foods are unhealthy, participant level of dietary restraint, the label of the apple, the perceived quality of the apple, and the perceived satiety of the apple. The regression coefficients of a model including these predictors of dessert choice are given (Table 2). None of these effects was found to interact with the label. Participant BMI, the perceived healthiness of the apple, and the apple’s perceived quality did not predict dessert choice. 

**Table 2:** Logistic regression model of the effect of label on apple selection .

	B	Std. Error	*P*	
Tasty	1.02	0.17	<0.001	***
Belief about taste and health				
Neutral	Ref			
Disagree	0.62	0.26	0.02	*
Agree	-0.61	0.27	0.03	*
Restraint (high vs. low)	0.47	0.24	0.05	*
Label				
No label	Ref			
‘Healthy’	0.01	0.33	0.99	
‘Succulent’	-0.07	0.32	0.84	
‘Healthy&Succulent’	0.79	0.34	0.02	*
‘Succulent&Healthy’	0.51	0.34	0.13	
Quality	-0.42	0.21	0.05	*
Filling	0.22	0.14	0.13	

* p<.05

*** p<.00

## Discussion

This study found that the intervention of a label combining the terms ‘healthy’ and ‘succulent’ to describe an apple increased the choice of the apple over the chocolate bar relative to when the apple was unlabelled. Contrary to our prediction, the same effect was not found when the single label ‘succulent’ was used. As predicted, the term ‘healthy’ did not increase the likelihood of the selection of an apple. 

Contrary to prediction, the effect of a label that included both terms - succulent and healthy - was found not to be mediated by the perceived tastiness of the apple. This observation is hard to explain with the available data for a number of reasons. First, the combined label may have increased the perceived tastiness of the apple, but the current study failed to detect this with the measure used. However, the observation that perceived tastiness of the apple did predict dessert choice independently of label, suggests that our measure of tastiness was sensitive to a property of the apple that is significant for behaviour. Second, the study may have been underpowered to detect such an effect. But given effects of label and perceived tastiness were found, but there was no association between these two, this seems unlikely. Third, the label may have been acting to increase apple selection by some other mechanism, which cannot be identified with the current data. 

We used the label ‘succulent’ to convey the quality of tastiness as opposed to simply ‘tasty’, because we perceived the former term to be more commonly applied to real-world marketing of fruit. It is possible, however, that this term suggests a quality of an apple that is quite different from taste. Alternatively, the combined labels may have been effective without altering perceived tastiness because on viewing the combined label, participants simultaneously brought to mind both health and taste. The combined label may therefore have been acting to bridge an associative gap that otherwise acts against choosing the apple. This gap comes about because choosing a given food primarily requries it to be considered tasty [3] but healthy foods, such as apples, are typically not thought of as tasty [6]. By explicitly stating both health and taste, the label may have acted as a simple prime to facilitate the simultaneous activation of both taste and health as attributes of the apple at the point of being considered in the dessert choice. Such a priming effect would enhance selection in favour of the apple without necessarily altering the attributes associated with apples or the strength of belief that healthy foods are seldom tasty.

Participants’ perceived tastiness of the apple and belief that tasty foods are unhealthy were the two strongest predictors of whether an apple was chosen over a chocolate bar, both being stronger predictors of choice than the label experienced and both acting independently of the label. 

The current study has a number of strengths and limitations. It used a simple experimental design, in which the effect of a single intervention – the label – on a simple and plausible real-world decision was assessed. The current study was also conducted entirely online, meaning participants knew no real food would be delivered following selection. This design makes the interpretation of the findings more straightforward than a correlational design looking at real-world food choices would have allowed, as many possible variables have been controlled for. One limitation, however, is that the current study did not allow for participants to actually consume their selected foods. While this could be a problem for some designs, there are a number of reasons why it might be less so in this case. Choosing foods does typically occur before consuming them, so in that sense the current task is realistic. All the foods used in the current study were chosen to be readily identifiable and highly familiar, allowing participants to bring to bear their own previous experience with consuming these foods. Thus while participants were not able to consume the foods as part of the study, they would have had available to them the same information as is often available when making a food choice. The only remaining concern is whether participants made a different selection in the current study to one they would have made had they known they would have had to consume the food. 

The recurring importance of perceived tastiness in selecting healthy foods and the link between taste and health suggests that these concepts and associations may be promising targets for future interventions. In other words, can strengthening the associations between health and taste for a given healthy food increase the extent to which healthy foods are chosen? Labelling may be one way to achieve this, and while only moderately effective in the current study, it has low associated costs and could have a large population-wide effects. Other interventions such as goal priming [12] or associative conditioning [13], while more complex to administer, may be more potent and would be interesting candidates for future research.
