# Correction: Choosing between an Apple and a Chocolate Bar: the Impact of Health and Taste Labels

**DOI:** 10.1371/annotation/12ecd041-f8c7-4615-a898-9ee60d2d78a7

**Published:** 2013-10-23

**Authors:** Suzanna E. Forwood, Alexander D. Walker, Gareth J. Hollands, Theresa M. Marteau

This article has been republished to replace Figure 1. If you downloaded the PDF previously, please download it again for the most up to date version. 

